# Tunnel collapse risk assessment based on improved quantitative theory III and EW-AHP coupling weight

**DOI:** 10.1038/s41598-022-19718-z

**Published:** 2022-09-26

**Authors:** Li Li, Bo Ni, Shixin Zhang, Yue Qiang, Zhongxu Zhang, Ling Zhou, Gang Liu, Longfei Cheng

**Affiliations:** School of Civil Engineering, Chongqing Three Gorges University, Wanzhou, Chongqing, 404100 China

**Keywords:** Engineering, Civil engineering

## Abstract

It is a multi-criteria decision issue to conduct a risk assessment of the tunnel. In this paper, a tunnel collapse risk assessment model based on the improved theory of quantification III and the fuzzy comprehensive evaluation method is proposed. According to the geological conditions and the construction disturbance classification method, the evaluation factors are selected, and the tunnel collapse risk level is divided into 5 levels according to the principle of maximum membership degree. The three groups of scores with the largest correlation ratio are calculated by the theory of quantification III to form the X, Y, and Z axes of the spatial coordinate system, The spatial distance of each evaluation factor is optimized by the Kendall correlation coefficient combined with the empirical formula, so that it can be used to judge the probability of the occurrence of the evaluation factor; taking the coupling of the objective entropy weight method (EW) and the subjective analytic hierarchy process (AHP) as the weight. Finally, the fuzzy comprehensive evaluation method is used to determine the possibility classification of tunnel collapse. Taking the Ka-Shuang water diversion tunnel as a case study, the comparison between the evaluation results of 10 tunnel samples and the status quo of the actual engineering area verifies the reliability of the method.

## Introduction

Due to the intricacy, concealment, and uncertainty of tunnel construction, safety accidents frequently occur during the procedure. Through statistical analysis of geological hazard events recorded in the tunnel system between 2002 and 2018, it was concluded that collapse was the main geological hazard during tunnel construction^1^. A tunnel collapse will not only increase construction difficulty, cost and the possibility of secondary disaster, but also endanger the safety of construction workers. Therefore, the assessment of tunnel collapse risk is a necessary measure to ensure the safe construction of tunnels.

Because a number of reasons can lead to tunnel collapse, the risk assessment of tunnels should be regarded as a multi-criteria issue^2^. Kim et al.^3^ deduced 14 influencing factors that lead to tunnel collapse during the process of construction from five aspects: geotechnical engineering characteristics, tunnel geometric conditions, groundwater conditions, excavation conditions, as well as support and reinforcement conditions. When establishing a comprehensive risk assessment system for tunnel collapse, large deformation of surrounding rock and mud scouring, Li et al.^4^ selected 12 influencing factors as comprehensive evaluation indicators from three aspects: engineering geological conditions, hydrological conditions, and construction methods. After analyzing typical tunnel collapse cases, Ou et al.^5^ selected 11 influencing factors as risk assessment system indicators from five aspects: engineering geological conditions, natural environment, design and construction, construction organization and management, and advanced geological forecasting. In this paper, considering the influence of geological conditions and construction disturbance, 11 influencing factors are selected as the evaluation factors for the collapse risk of the northern water diversion tunnel with reference to the method of Zhai et al.^9^.

For multi-criteria problems, the determination of weight in traditional methods is very important. Chu^6^ and Dai^7^ used the AHP method to reasonably distribute the weights of the factors affecting the safety of tunnel construction, and used the membership function and the exponential weight to gradually calculate the corresponding risk level. The weight determination process of this method relies on expert experience, and the membership function classification is subjective. Gao et al.^8^ established a comprehensive risk assessment model for tunnel collapse based on entropy weight and grey correlation degree. Zhai et al.^9^ used the entropy weight method and the analytic hierarchy process combined with the undetermined measure theory to determine the multi-index comprehensive evaluation vector, and evaluated the tunnel collapse risk according to the principle of maximum membership degree. Both Gao and Zhai combine objective and subjective methods to determine weights, which are further improved compared to the former's evaluation reliability. With the development of theories about artificial intelligence, the Artificial Neural Networks (ANNs) are used to predict the stability conditions of roadways^10^. He et al.^11^ combined the Interpreted Structure Modeling (ISM) and the Fuzzy Bayesian Networks (FBN), where the Fuzzy Bayesian Networks (FBN) obtain the prior probability and conditional probability of the node by aggregating the opinions of experts, using the Similarity Aggregation Method (SAM), respectively, to determine the hierarchical relationship and the interaction strength of each risk factor for risk analysis. Mahdevari et al.^12^ found that the Particle Swarm Optimization (PSO) algorithm can significantly improve the performance of the Adaptive Neuro-Fuzzy Inference System (ANFIS), so the PSO-ANFIS model was proposed to predict unstable areas of underground roads. Zhou et al.^13^ optimized the Support Vector Machine (SVM) of the machine learning model through the Whale Optimization Algorithm (WOA), and established a WOA-SVM model to classify the extrusion behavior of the tunnel surrounding rock. In addition to the evaluation methods in the above section, Cloud model^14,15^, interval risk assessment^16^, event tree analysis^17^, fault tree analysis^18^, BP neural network^19^ are also used in tunnel and underground engineering risk assessment. To sum up, the current risk assessment of tunnels is usually a combination of subjective and objective methods. Although some studies have shown that grading standards are also ambiguous, the ambiguity of evaluation factors is the main factor^20^.

Therefore, this paper selects a branch of multivariate analysis "The Theory of Quantification III" as the basic theory for determining the weight of evaluation factors. In contrast to the qualitative or quantitative benchmark variables of Theory of Quantification I and II, the Theory of quantification III is a method that combines multiple qualitative and quantitative data to establish a comprehensive evaluation model^21–23^, with the advantages of applicability and objectivity, that is currently rarely used in the natural sciences. After calculating the weights of the evaluation factors, combined with the "normal distribution" membership function of the Fuzzy Comprehensive Evaluation Method, the tunnel collapse risk is classified. Section “Introduction” introduces the process of constructing the evaluation model by combining the Improved Theory of Quantification III with the Fuzzy Comprehensive Evaluation Method; Section “Build the model” applies the model to engineering examples and analyzes the evaluation results; Section “Example verification” discusses the rationale for model construction and its mathematical implications.

## Build the model

### The improved theory of quantification III

#### General calculation method

The Theory of Quantification III is based on a reflection matrix constructed by dividing qualitative or quantitative data into several disjoint intervals, and assigning an appropriate value *b*_*j*_ (*j* = 1,2,…,*m*) to each category, which is called the category score, so that categories with similar response situations have similar scores; At the same time, a corresponding value *y*_*i*_ (*1*,2,…,*n*) is also assigned to each sample, which is called the sample score, so that samples with similar reaction conditions have similar scores. In this way, the score *b*_*j*_ (or *y*_*i*_) has inherent meaning as a quantitative representation of categories (or samples), so it can comprehensively express the relationship between categories (or samples), and analyze the dominant factors in the variables.

In the Theory of Quantitative, qualitative variables are called items, and the different intervals that each item is divided into are called categories. Suppose there are s variables in total, of which there are m classified items, the *j-*th item has *r*_*j*_ categories, and a total of $r = \sum\limits_{{}}^{{}} {r_{j} \left( {1 \le j \le m} \right)}$ categories. From this, *n* sample data can be constructed into a reflection matrix *X* with *n* rows and *r* + *s* columns:

1 $$ X = \left( {\begin{array}{*{20}c}    {\delta _{{1\left( {1,1} \right)}} } &  \cdots  & {\delta _{{1\left( {1,r_{1} } \right)}} } &  \cdots  & {\delta _{{1\left( {m,1} \right)}} } &  \cdots  & {\delta _{{1\left( {m,r_{m} } \right)}} } & {u_{{11}} } &  \cdots  & {u_{{1s}} }  \\    {\delta _{{2\left( {1,1} \right)}} } & {} & {\delta _{{2\left( {1,r_{1} } \right)}} } & {} & {\delta _{{2\left( {m,1} \right)}} } & {} & {\delta _{{2\left( {m,r_{m} } \right)}} } & {u_{{21}} } & {} & {u_{{2s}} }  \\     \vdots  &  \cdots  &  \vdots  &  \cdots  &  \vdots  &  \cdots  &  \vdots  &  \vdots  &  \cdots  &  \vdots   \\    {\delta _{{n\left( {1,1} \right)}} } & {} & {\delta _{{n\left( {1,r_{1} } \right)}} } & {} & {\delta _{{n\left( {m,1} \right)}} } & {} & {\delta _{{n\left( {m,r_{m} } \right)}} } & {u_{{n1}} } & {} & {u_{{ns}} }  \\   \end{array} } \right) $$

In the Eq. (1):

2 $$ \delta_{i} \left( {j,r_{j} } \right) = \left\{ \begin{gathered} 1\left( {{\text{When\;the\;qualitative\;data\;of\;item\;}}j\;{\text{in\;the\; }}i{\text{th\;sample\;is\;}}r_{j}\;{\text{\;category}}} \right) \hfill \\ 0\left({{\text{When\;the\;qualitative\;data\;of\;item\;}}j\;{\text{in\;the\;}}i{\text{th\;sample\;is\;not\;the\;}}r_{j}\;{\text{\;category}}} \right) \hfill \\ \end{gathered} \right. $$

In the Eq. (2): $\delta_{i} \left( {j,r_{j} } \right)$ represents the response on the *r*_*j*_ category of the *i*-th sample *j* item, and $u\left( {i,k} \right)$ represents the *k*-th quantitative variable’s response value in the *i*-th sample.

In the analysis method of Theory of Quantification III, the total *r* + *s* dimension category is assigned a score in the form of:

3 $$  b = \left\{ {b_{{11}} , \ldots b_{{1r}} , \ldots ,b_{{m1,}} , \ldots ,b_{{mr_{m} }} , \ldots ,a_{1} , \ldots ,a_{s} } \right\}^{T}  $$

Average score for the *i*-th sample:

4 $$ y_{i} = \frac{1}{m + s}\left( {\sum\limits_{j = 1}^{m} {\sum\limits_{k = 1}^{{r_{j} }} {b_{jk} \delta \left( {j,k} \right) + \sum\limits_{k = 1}^{s} {a_{i} u_{ik} } } } } \right) $$

Therefore, the main problem of the Theory of Quantification III is transformed into solving vector *b*_*j*_ (relationship between categories) and vector *y*_*i*_ (relationship between samples). The specific solution process is as follows:

1. Record the sample score as $Y = \left\{ {y_{1} ,y_{2} , \cdots ,y_{n} } \right\}^{T}$, the sum of each sample’s responses on the *j* item *k* category is $g_{jk} = \sum\limits_{i = 1}^{n} {\delta_{i} \left( {j,k} \right)}$, sample score $Y = \left\{ {y_{1} ,y_{2} , \cdots ,y_{n} } \right\}^{T} = \frac{1}{m + s}Xb$, then the overall mean of the *n* sample scores is:
  5 $$ \overline{y} = \frac{1}{{n\left( {m + s} \right)}}g^{T} b $$
2. Considering each sample as a group, the between-group variance is:
  6 $$ \sigma_{b}^{2} = \frac{1}{n}\sum\limits_{i = 1}^{n} {\left( {y_{i} - \overline{y} } \right)^{2} } = \frac{1}{{n(m + s)^{2} }}b^{T} Hb $$
  In the Eq. (6):
  $$ H = X^{T} X - \frac{1}{n}gg^{T} $$
  From Eq. (6), we can get:
  $$ n\left( {m + s} \right)^{2} \sigma_{b}^{2} = b^{T} Hb $$
3. The total variance of the sample is:
  7 $$ \sigma^{2} = \frac{1}{{n\left( {m + s} \right)}}\left( {\sum\limits_{j = 1}^{m} {\sum\limits_{k = 1}^{{r_{j} }} {b_{jk}^{2} g_{jk} + n\sum\limits_{i = 1}^{s} {a_{i}^{2} } } } } \right) - \overline{y}^{2} = \frac{1}{{n\left( {m + s} \right)}}b^{T} Lb $$
  Among them, *G* is a diagonal matrix of order *r* + *s*:
  $$ L = G - \frac{1}{{n\left( {m + s} \right)}}gg^{T} $$
4. The correlation ratio between sample group variance and total variance is:
  8 $$ \eta^{2} = \frac{{\sigma_{b}^{2} }}{{\sigma^{2} }} = \frac{{b^{T} Hb}}{{\left( {m + s} \right)b^{T} Lb}} $$

To maximize the correlation ratio and satisfy the constraints $b^{T} Lb = 1$, $g^{T} b = 0$, the expression for solving the vector *b* is:

9 $$ Hb = \lambda \left( {m + s} \right)Lb $$

where λ represents the eigenvalue of the equation.

Both the category score *b* and the sample score *yi* obtained from this are one-dimensional, and its geometric meaning refers to that the feature vector *b* is regarded as a factor axis, and the sample score vector *yi* is regarded as the projection on this axis. The corresponding largest eigenvalue indicates the direction in which this axis gives the projection the greatest degree of dispersion. Therefore, when the one-dimensional representation effect is not ideal, the eigenvector *b* corresponding to the largest top k eigenvalues $\lambda_{1} \ge \lambda_{2} \ge \cdots \ge \lambda_{k} > 0$ can be selected to classify the categories^21^.

#### Improvement steps

The weights calculated by the quantitative theory mainly consider the frequency of occurrence of each factor and the internal meaning of the scores *y*_*i*_ of each sample. However, in practical engineering, the influence of the correlation between the categories *b*_*i*_ of each evaluation factor cannot be ignored^24–28^. Considering that the quantitative theory has more detailed classification scores for various categories, we use the Kendall algorithm to calculate the correlation between the factors. The core idea is to calculate the number of different pairs between two ordered sets. The calculation process is as follows:

Suppose that there are *N* objects in a group of *φ*:

10 $$ \varphi = \left\{ {a,b, \ldots ,x,y} \right\} $$

An ordered set of *N* objects can be decomposed into an ordered pair of $1/2N\left( {N - 1} \right)$, for example $\varphi = \left\{ {a,b,c,d} \right\}$ then:

11 $$ \varphi_{1} = \left\{ {\left[ {a,c} \right],\left[ {a,b} \right],\left[ {a,d} \right],\left[ {c,b} \right],\left[ {c,d} \right],\left[ {b,d} \right]} \right\} $$

The difference distance between the two ordered pairs *φ*_*1*_ and *φ*_*2*_ is denoted as $d_{\Delta } \left( {\varphi_{1} ,\varphi_{2} } \right)$.

Hence the following Eq. (12) for the Kendall correlation coefficient^29^. It is given:

12 $$ \tau = 1 - \frac{{2 \times \left[ {d_{\Delta } \left( {\varphi_{q} ,\varphi_{2} } \right)} \right]}}{{N\left( {N - 1} \right)}} $$

i.e.$\tau = P(same) - P(diffreent)$.where *τ* represents the difference between the probability that a pair of randomly obtained objects are in the same order and the probability that they are in a different order.

In this paper, a correlation (*τ* ≥ 0.5) is assumed between the two influencing factors whose correlation degree is greater than 0.5. To this end, the correlation feedback is used to adjust the spatial distance (i.e. the weight), and according to the Rocchio equation^30^, we propose the following update function:

13 $$ \kappa_{f}^{\prime } = \kappa_{f} + \frac{\beta }{{\left| {cases_{REL} } \right|}}\sum\limits_{{b_{i} \in cases_{REL} }}^{{}} {b_{i} } - \frac{\gamma }{{\left| {cases_{NR} } \right|}}\sum\limits_{{b_{j} \in cases_{NR} }}^{{}} {b_{j} } $$

Among them, casesREL represents a group that is correlated with a certain evaluation factor *U*_*i*_, and casesNR is a group that is not correlated with a certain evaluation factor *Ui*. *b*_*i*_ and *b*_*j*_ take values in the set of scores with the largest correlation ratio in the quantitative theoretical calculation results. In practice, *γ* is often taken as 0.25, and *β* is often taken as 0.75^30^.

### Coupling weights

The Entropy Weight Method (EW) and the Analytic Hierarchy Process (AHP) are commonly used objective and subjective weighting methods in Multiple Criteria Decision Making^31–33^. In this paper, the multiplicative synthesis normalization method is used to determine the EW-AHP coupling weight as the initial weight.

The Entropy Weight Method (EW)^34^ uses the entropy value *g*_*ij*_ to measure the amount of information. Assuming that the evaluation index *Ui* is equivalent to the importance of other indicators, it is represented by $\omega \left( {0 \le \omega_{ij} \le 1,\sum\limits_{j = 1}^{m} {\omega_{ij} = 1} } \right)$, then $\omega_{ij}$ is the weight of the evaluation factor *Ui*. The specific calculation of Eq is as follows:

1. Perform a dimensionless processing on x_ij_ to get $x_{ij}^{^{\prime}}$:
  14 $$ \begin{gathered} {\text{Positive indicator}}:x_{ij}^{\prime } = \frac{{x_{ij} - \min \left( {x_{ij} } \right)}}{{\max \left( {x_{ij} } \right) - \min \left( {x_{ij} } \right)}} + \alpha \hfill \\ {\text{Inverse indicator}}:x_{ij}^{\prime } = \frac{{\min \left( {x_{ij} } \right) - x_{ij} }}{{\max \left( {x_{ij} } \right) - \min \left( {x_{ij} } \right)}} + \alpha \hfill \\ \end{gathered} $$
  In the equation, $\min \left( {x_{ij} } \right)$ is the minimum value and $\max \left( {x_{ij} } \right)$ is the maximum value. In order to eliminate the influence of 0 value, add a minimum value close to the value of $x_{ij}^{^{\prime}}$ after the dimensionless processing to translate. In this paper, $\alpha$ is taken as 0.001.
2. Perform a standardized processing, P_ij_ refers to the proportion of the *j*-th index of the *i*-th sample in the overall data:
  15 $$ P_{ij} = {{x_{ij}^{*} } \mathord{\left/ {\vphantom {{x_{ij}^{*} } {\sum\limits_{i = 1}^{n} {x_{ij}^{*} } }}} \right. \kern-\nulldelimiterspace} {\sum\limits_{i = 1}^{n} {x_{ij}^{*} } }} $$
3. Calculate the difference coefficient g_i_ of the *j*-th index:
  16 $$ g_{j} = 1 + \frac{1}{\ln n}\sum\limits_{i = 1}^{n} {p_{ij} \ln p_{ij} } $$
4. Calculate the weight $\omega_{j}$ of the *j*-th index:
  17 $$ \omega_{j} = {{g_{j} } \mathord{\left/ {\vphantom {{g_{j} } {\sum\limits_{j = 1}^{m} {g_{j} } }}} \right. \kern-\nulldelimiterspace} {\sum\limits_{j = 1}^{m} {g_{j} } }} $$

The Analytic Hierarchy Process (AHP) method determines the subjective weight of the evaluation index. Firstly, the identified risk factors causing tunnel collapse are compared in pairs through expert judgment according to the 9-level evaluation method^35^ to form a judgment matrix *S*, and the consistency test is carried out. Then the judgment matrix is calculated by the square root method to obtain the weight of each factor^33^.

1. Constructing the judgment matrix:
  18 $$ M = \left( {\begin{array}{*{20}c} {b_{1} } & \ldots & {b_{j} } \\ \vdots & \ddots & \vdots \\ {b_{j} } & \cdots & {b_{j} } \\ \end{array} } \right)_{j \times j} $$
  b_j_ represents the importance level determined by the 9-level evaluation method^40^. By calculating the maximum eigenvalue of the judgment matrix M, the normalized eigenvector is obtained, and the the weight value of each index is obtained too.
2. In order to verify whether the importance level assigned to risk indicators is reasonable, the consistency test method of Wang et al.^36^ is used.
  19 $$ CI = \frac{{\lambda_{\max } - n}}{n - 1} $$
  20 $$ CR = CI/RI $$
  In the equation, CI is the consistency index, RI is the random consistency index^36^, and CR is the consistency ratio.

The composite weight is the coupling of objective data and subjective experience. In this paper, the multiplicative synthesis normalization method is used to calculate the coupling weight of the evaluation factor, and the EW-AHP coupling weight is used as the initial weight. After calculating the initial weight, combine the previous TQ-III weight into the following Eq. (21) to calculate the final weight:

21 $$ \omega_{j} = \left( {\alpha_{j} .\beta_{j} } \right)/\sum\limits_{j = 1}^{m} {\left( {\alpha_{j} .\beta_{j} } \right)} $$

In the Eq. (21): $\omega_{j}$ is the comprehensive weight of the *j*-th evaluation factor; $\alpha_{j}$ and $\beta_{j}$ are the TQ-III and EW-AHP weights of the *j*-th evaluation factor, and *m* is the number of evaluation factors.

### Fuzzy comprehensive evaluation

Considering that the evaluation of tunnel collapse possibility is affected by the uncertainty of many factors, the application of the Fuzzy Comprehensive Evaluation Method can often achieve better practical results in tunnel collapse risk evaluation. In this method, the membership degree is calculated through the mapping of the factor set to the comment set, and then combined with the weight calculated by the Theory of Quantification III, a relatively objective fuzzy comprehensive evaluation result can be obtained.

Common membership function forms include "trapezoid", "semi-trapezoid", "normal", "k-th parabolic", "Cauchy", "Γ", "ridge" and so on^37^. Considering that the evaluation factors in this paper are scattered, the "normal" type membership function is selected to calculate the membership degree of the factor set *U* to the comment set *V*^38^. In the risk level classification of this paper, level *I* belongs to a small fuzzy distribution, levels *II*, *III*, and *IV* belong to an intermediate fuzzy distribution, and level *V* belongs to a large fuzzy distribution. The formula of the smaller membership function designed according to the normal distribution is:

22 $$ A_{{\text{I}}} \left[ {x_{j} \left( i \right)} \right] = \left\{ \begin{gathered}   1,x_{j} \left( i \right) \le a_{1}  \hfill \\   \exp \left[ { - \left( {\frac{{x_{j} \left( i \right) - a}}{\sigma }} \right)^{2} } \right] \hfill \\  \end{gathered}  \right.,x_{j} \left( i \right) > a_{1}  $$

The three intermediate membership functions are:

23 $$ A_{{{\text{II}}}} \left[ {x_{j} \left( i \right)} \right] = \exp \left[ { - \left( {\frac{{x_{j} \left( i \right) - a_{2} }}{\sigma }} \right)^{2} } \right] $$

24 $$ A_{{{\text{III}}}} \left[ {x_{j} \left( i \right)} \right] = \exp \left[ { - \left( {\frac{{x_{j} \left( i \right) - a_{3} }}{\sigma }} \right)^{2} } \right] $$

25 $$ A_{{{\text{IV}}}} \left[ {x_{j} \left( i \right)} \right] = \exp \left[ { - \left( {\frac{{x_{j} \left( i \right) - a_{4} }}{\sigma }} \right)^{2} } \right] $$

The larger membership function is:

26 $$ A_{{\text{V}}} \left[ {x_{j} \left( i \right)} \right] = \left\{ \begin{gathered}   0,\;x_{j} \left( i \right) \le a_{5}  \hfill \\   1 - \exp \left[ { - \left( {\frac{{x_{j} \left( i \right) - a}}{\sigma }} \right)^{2} } \right],\;x_{j} \left( i \right) > a_{5}  \hfill \\  \end{gathered}  \right.  $$

In the formula, *A* represents the membership degree of the *i*-th factor of the *j*-th sample at a certain level, $x_{j} \left( i \right)$ represents the measured data of the *i-*th factor of the *j*-th sample. where *a*_*i*_ is the location parameter of the normal distribution and *σ* is the shape parameter describing the degree of dispersion of the normal distribution. The formula is as follows.

27 $$ a_{i} = \frac{1}{{2\left( {e_{i} + e_{i + 1} } \right)}} $$

28 $$ \sigma = 0.6\left( {e_{i + 1} - e_{i} } \right) $$

*e*_*i*_ and *e*_*i*+*1*_ are the upper and lower limits of a certain risk level evaluation factor.

The fuzzy judgment matrix calculated by the membership function is:

29 $$ B = \left( {\begin{array}{*{20}c} {b_{11} } & \ldots & {b_{1v} } \\ \vdots & \ddots & \vdots \\ {b_{u1} } & \cdots & {b_{uv} } \\ \end{array} } \right)_{u \times v} $$

In the Eq. (29): *u* is the evaluation factor; *v* is the risk level; *b*_*uv*_ represents the membership degree of the *u*-th evaluation factor to the risk level *v*.

After obtaining the comment set, weight set, and single-factor judgment matrix, make a fuzzy linear transformation on the fuzzy judgment matrix and turn the weight set into a fuzzy subset of the comment set:

30 $$ A = W \times B $$

**Figure 1:**
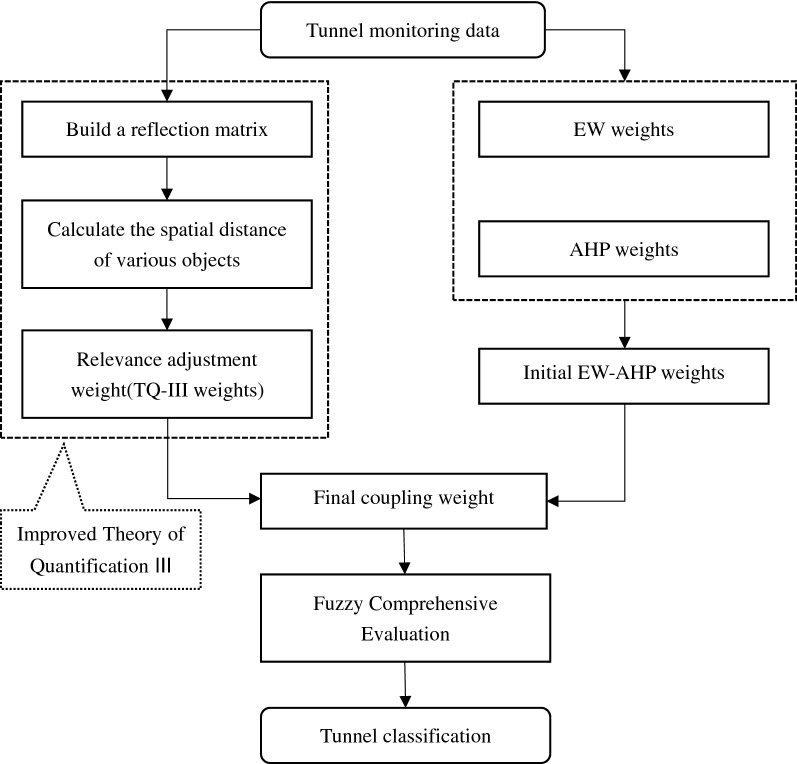
Model building flowchart.

In the Eq. (30): *W* is the coupling weight of Sect. “Coupling weights” (Fig. 1).

As shown in Fig. 1, the first step is to construct a reflection matrix from the Theory of Quantification III, and calculate the scores of various objects. Then select the three groups of scores with the largest correlation ratio to form the *X*, *Y*, and *Z* axes, calculate the spatial distance *κ* of each category and, the Kendall correlation coefficient based on *κ*, then optimize the coefficient with the Rocchio equation to obtain the weight of each influencing factor (ie TQ-III weight); The second step is to calculate the multiplicative synthesis normalized weight of EW-AHP as the initial weight, which is coupled with the TQ-III weight so that the weight can be dynamically adjusted with the characteristics of the project area; The third step uses the Fuzzy Comprehensive Evaluation Method of the normal distribution membership function to classify the possibility of tunnel collapse.

## Example verification

### Overview of research object’sproject and geology

#### Overview of the project

The second phase of the Beijiang Water Supply Project consists of three diversion tunnels: West-Second, Ka-Shuang, and Double-Third. The total length of the tunnels is 516.2 km, of which the Ka-Shuang tunnel is 283.3 km long, making it the longest water delivery tunnel in the world. The average burial depth is 428 m, and the maximum burial depth is 774 m, which is a no-pressure diversion tunnel. The diameter of the cavern excavated by drilling and blasting is 6.64–7.4 m, and the diameter of the cavern excavated by TBM is 7.1 m. The surrounding rock of the cavern is mainly grade II and grade III (According to the relevant standards of Chinese tunnel construction^44^, the quality of surrounding rock is divided into 6 grades from good to poor, and grades II and III are hard rocks with better comprehensive quality), accounting for 86.2%, and the saturated compressive strength is mostly between 50 and 140 MPa^39^.

#### Topography and physiognomy

The project is located in the hilly area and low mountain area between the southern slope of the Altai Mountains and the northern slope of the East Tianshan Mountains, with an altitude of 750–1300 m, the terrain is undulating, the slope of the mountain is gentle, and the bedrock is mostly exposed, the physiognomy is mainly desert^39^.

#### Stratigraphic lithology

The strata in this area are dominated by the ancient strata of the Devonian and Carboniferous, followed by granite, and very few areas are Permian and Triassic strata. Among them, the Devonian and Carboniferous tuffs, tuffaceous sandstones and calcareous sandstones strata have a total length of 209.1 km, accounting for 73.8% of the tunnel length; the total length of the biotite granite and granodiorite strata intruded in the late Hercynian is 59.6 km, accounting for 21% of the tunnel length; the mudstone, sandstone and glutenite of the Permian and Triassic have a total length of 12.1 km, accounting for 4.3% of the tunnel length^39^.

#### Geological structure

**Figure 2:**
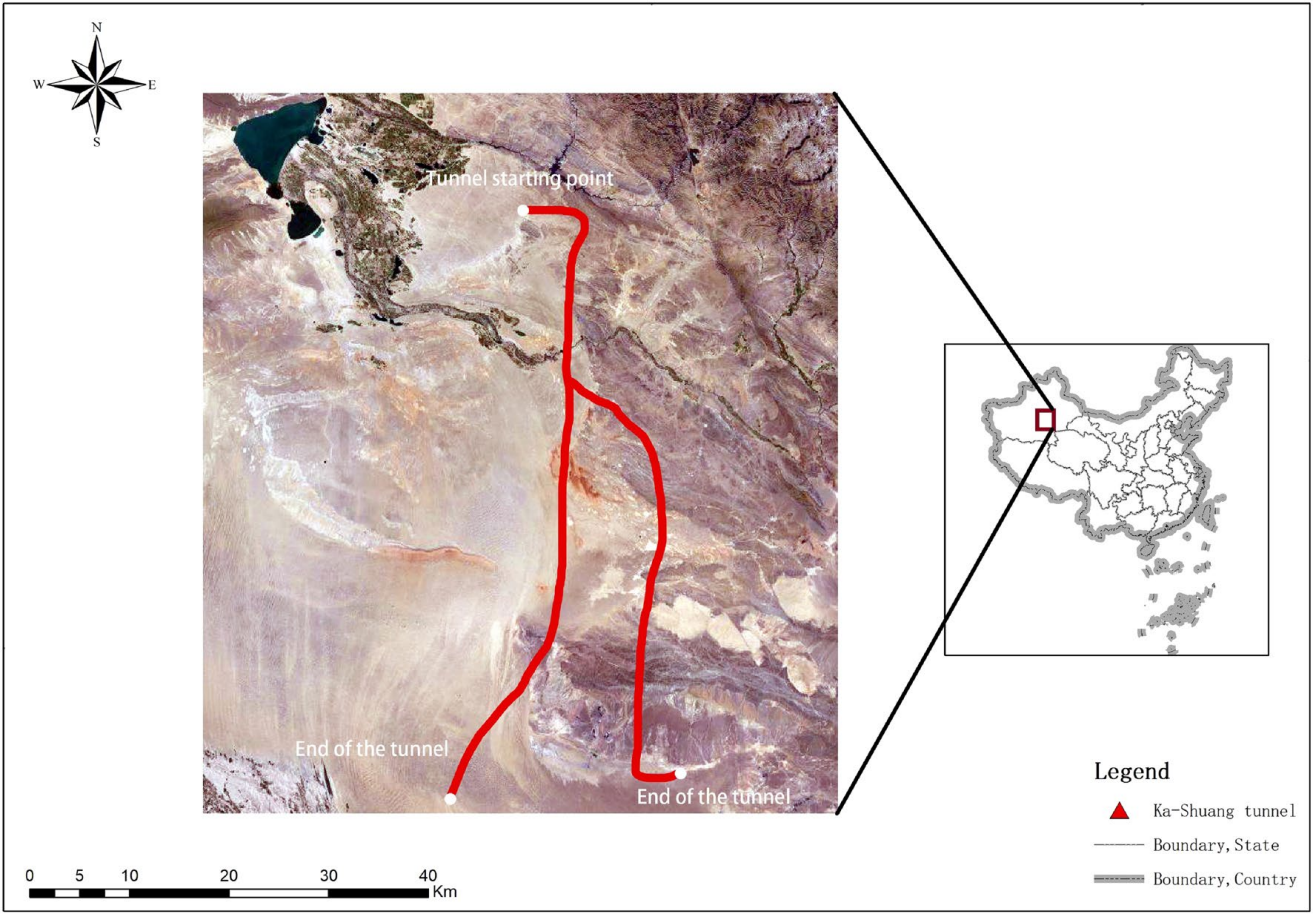
Location of the Ka-Shuang Tunnel (Arcgis10.8 https://www.esri.com/en-us/arcgis/products/mapping/overview).

The project area is located in the two major tectonic unit intervals of the Altai fold system and the Junggar-North Tianshan fold system. Due to the influence of the fold structure on the topography and geological structure, a series of compressive faults and compressive torsional faults have developed in this area. There are 5 regional fault zones with obvious structural traces on the surface, 72 secondary faults, and the general width of the fracture zone is 10–30 m. Through the comprehensive analysis of drilling core exposure, downhole TV snooping, geophysical sound wave testing and other methods. It is found that the faults and fissures near the tunnel do not developed, and the fissures are dominated by medium-steep dips. The surface water in the project area is lacking, and the groundwater is mainly composed of a small amount of bedrock fissure water. The water quality is poor, and it is corrosive to concrete and steel bars in concrete structures^39^. In this paper, 10 typical sections are selected as the evaluation objects in the research area (Fig. 2).

By summarizing the existing research results, the factors that cause tunnel collapse are mainly divided into geological condition factors, construction factors and design factors^40^. Refering to the statistical analysis results of Zhu Jie et al.^2^, on 242 road tunnels, 104 railway tunnels, 35 hydraulic tunnels, a total of 381 effective collapse cases, the main influencing factors are the grade of surrounding rock (18.35%), groundwater (11.15%), rainfall (10.19%), supporting method (7.29%), fractured and broken zone (6.90%), the integrity of rock mass (6.13%), tunnel Buried depth (4.39%). Among them, the rainfall factor is generally considered in the shallow buried section or the opening, so it is not considered in this paper.

**Figure 3:**
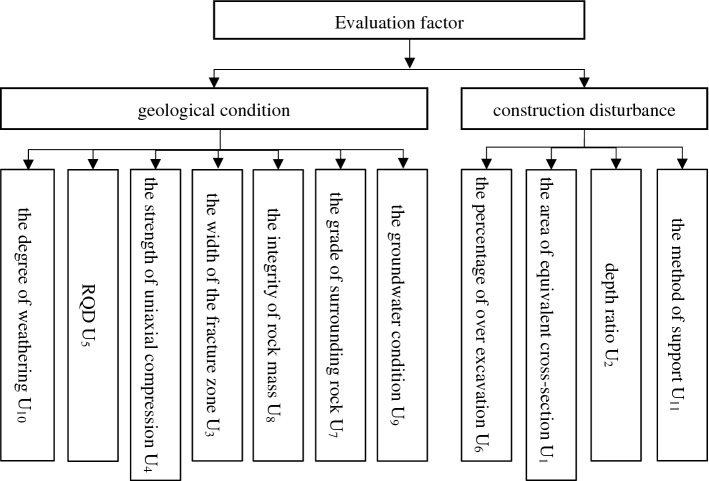
Evaluation index hierarchy diagram.

In summary, combined with the literature induction in the introduction chapter. According to the geological conditions and the classification method of construction disturbance, this paper selects the area of equivalent cross-section *U*_1_, depth ratio *U*_2_ (the ratio of tunnel buried depth to diameter), the width of the fracture zone *U*_3_ (the width of a section of strongly fragmented rock caused by a fault or a dense zone of fissures^2^), the strength of uniaxial compression *U*_4_, RQD *U*_5_ (rock quality index, reflecting the geological conditions of the stratum where the tunnel is located), the percentage of over excavation *U*_6_, the grade of surrounding rock *U*_7_ (the tunnel surrounding rock grade determined by the longitudinal wave), the integrity of rock mass *U*_8_ (the ratio of the rock mass elastic longitudinal wave to the rock elastic longitudinal wave), the groundwater condition *U*_9_, the degree of weathering *U*_10_ and the method of support *U*_11_ are used as tunnel collapse risk assessment factors (Fig. 3).

The tunnel-related survey data^9^ in Table 1 are used as the basic data of Quantitative Theory III and the basic data of the decision matrix of the fuzzy comprehensive evaluation method (Table 1).

**Table 1:** Tunnel monitoring data.

Serial number	U_1_(/m^2^)	U_2_	U_3_(/m)	U_4_(/MPa)	U_5_(%)	U_6_(%)	U_7_	U_8_	U_9_	U_10_	U_11_
S1	47.8	8.97	0	75	0.81	0.75	1.8	0.8	None	Unweathered	Steel arch
S2	47.8	2.5	25	15	0.22	1.2	1.4	0.3	Linear	Strong weathering	Steel arch
S3	47.8	1.8	20	40	0.45	1.04	1.6	0.55	Drip	Moderate weathering	Steel arch
S4	38.5	28.4	0	55	0.88	0.85	1.7	0.8	Linear	Slightly weathered	Anchor
S5	38.5	17.3	0	101	0.71	0.9	3.8	0.62	Influx	Unweathered	Anchor
S6	38.5	27.2	15	55	0.45	1.25	2.4	0.8	Drip	Moderate weathering	Anchor
S7	23.7	20	0	140	0.7	0.8	4	0.7	Drip	Unweathered	Loop excavation
S8	23.7	22.4	15	4	0.14	1.15	1.2	0.2	None	Moderate weathering	Loop excavation
S9	23.7	27.5	0	108	0.771	1.02	3.8	0.9	None	Slightly weathered	Loop excavation
S10	23.7	27.6	40	55	0.41	1.05	2.4	0.4	Damp	Slightly weathered	Loop excavation

In this paper, referring to Zhai et al.^9^ and Hyu et al.^43^ classification standard of tunnel collapse risk factors, a comment set *V* = {*v*_1_, *v*_2_, *v*_3_, *v*_4_, *v*_5_} is formulated with five risk levels, which are grade *I* (no risk) and grade *II* (slight risk), grade *III* (high risk), grade *IV* (slight collapse), and grade *V* (severe collapse) (Table 2).

**Table 2:** Tunnel classification.

Risk level	U_1_(/m^2^)	U_2_	U_3_(/m)	U_4_(/MPa)	U_5_(%)	U_6_(%)	U_7_	U_8_	U_9_	U_10_	U_11_
I	< 20	> 7	none	> 150	90 ~ 100	< 100	> 4.5	0.9 ~ 1	None	Unweathered	None
II	20 ~ 45	4.5 ~ 7	0 ~ 20	100 ~ 150	75 ~ 90	100 ~ 105	3.5 ~ 4.5	0.75 ~ 0.9	Moist or dripping	Slightly weathered	Shotcrete
III	45 ~ 70	2.5 ~ 4.5	20 ~ 30	50 ~ 100	50 ~ 75	105 ~ 110	2.5 ~ 3.5	0.5 ~ 0.75	Rain-like	Moderate weathering	Anchor
IV	70 ~ 120	1 ~ 2.5	30 ~ 50	10 ~ 50	25 ~ 50	110 ~ 120	1.5 ~ 2.5	0.2 ~ 0.5	Linear	Strong weathering	Steel arch
V	> 120	< 1	> 50	< 10	< 25	> 120	< 1.5	0 ~ 0.2	Influx	Fully weathered	Loop excavation

### The improved theory of quantification III

According to the grading standard in Table 2, the Theory of Quantification III is used to calculate and determine the main controlling factors affecting tunnel collapse. The 11 projects have 5 sub-categories respectively, and the total number of categories is 55. After calculation, the first three largest eigenvalues *λ*_1_ = 0.124, *λ*_2_ = 0.090, *λ*_3_ = 0.078 and their corresponding correlation ratios are 41, 18, and 28%, the sum has reached 87%, which can largely represent the information of influencing factors. The maximum eigenvalue and corresponding category scores are shown in Table 3.

**Table 3:** Influencing factor category scores.

Influencing factors		Influencing factor number	b_1_ (The correlation ratio is 41%)	b_2_ (The correlation ratio is 18%)	b_3_ (The correlation ratio is 28%)
The area of equivalent cross-section U_1_(/m^2^)	< 20	1	− 0.0704	− 0.0389	− 0.0499
	20 ~ 45	2	− 0.0675	0.0494	− 0.0257
	45 ~ 70	3	0.1576	− 0.1153	0.0600
	70 ~ 120	4	− 0.0704	− 0.0389	− 0.0499
	> 120	5	− 0.0704	− 0.0389	− 0.0499
Depth ratio U_2_	> 7	6	− 0.0633	0.0327	− 0.0226
	4.5 ~ 7	7	− 0.0704	− 0.0389	− 0.0499
	2.5 ~ 4.5	8	− 0.0704	− 0.0389	− 0.0499
	1 ~ 2.5	9	0.2533	− 0.1308	0.0903
	< 1	10	− 0.0704	− 0.0389	− 0.0499
The width of the fracture zone U_3_(/m)	none	11	− 0.1282	− 0.0373	0.0800
	0 ~ 20	12	0.0947	0.1312	− 0.1333
	20 ~ 30	13	0.3507	− 0.1683	0.2888
	30 ~ 50	14	0.0064	− 0.0387	− 0.2889
	> 50	15	− 0.0704	− 0.0389	− 0.0499
The strength of uniaxial compression U_4_(/MPa)	> 150	16	− 0.0704	− 0.0389	− 0.0499
	100 ~ 150	17	− 0.1792	− 0.0054	0.1665
	50 ~ 100	18	− 0.0229	− 0.0490	− 0.1900
	10 ~ 50	19	0.2533	− 0.1308	0.0903
	< 10	20	0.1225	0.4739	0.0798
RQD U_5_(%)	90 ~ 100	21	− 0.0704	− 0.0389	− 0.0499
	75 ~ 90	22	− 0.0821	− 0.0389	− 0.0158
	50 ~ 75	23	− 0.1974	− 0.0349	0.2237
	25 ~ 50	24	0.0559	− 0.0397	− 0.2562
	< 25	25	0.2366	0.1528	0.1843
The percentage of over excavation U_6_(%)	< 100	26	− 0.1246	− 0.0601	0.0869
	100 ~ 105	27	0.0066	− 0.0261	− 0.1150
	105 ~ 110	28	− 0.0704	− 0.0389	− 0.0499
	110 ~ 120	29	0.2366	0.1528	0.1843
	> 120	30	0.0055	0.0130	− 0.3714
The grade of surrounding rock U_7_	> 4.5	31	− 0.0704	− 0.0389	− 0.0499
	3.5 ~ 4.5	32	− 0.1792	− 0.0054	0.1665
	2.5 ~ 3.5	33	− 0.0704	− 0.0389	− 0.0499
	1.5 ~ 2.5	34	0.0692	− 0.0763	− 0.0966
	< 1.5	35	0.1225	0.4739	0.0798
The integrity of rock mass U_8_	0.9 ~ 1	36	− 0.1427	0.0537	0.0522
	0.75 ~ 0.9	37	− 0.0327	− 0.0525	− 0.1570
	0.5 ~ 0.75	38	− 0.0796	− 0.0544	0.1131
	0.2 ~ 0.5	39	0.1785	− 0.1035	− 0.0001
	0 ~ 0.2	40	0.1225	0.4739	0.0798
The groundwater condition U_9_	none	41	− 0.0180	0.1478	0.0438
	Moist or dripping	42	− 0.0026	− 0.0339	− 0.1458
	Rain-like	43	− 0.0704	− 0.0389	− 0.0499
	Linear	44	0.1405	− 0.1272	0.0948
	Influx	45	− 0.2165	− 0.0531	0.2621
The degree of weathering U_10_	None	46	− 0.1429	− 0.0514	0.1490
	Slightly weathered	47	− 0.0687	− 0.0237	− 0.1119
	Moderate weathering	48	0.0947	0.1312	− 0.1333
	Strong weathering	49	0.3507	− 0.1683	0.2888
	Fully weathered	50	− 0.0704	− 0.0389	− 0.0499
The method of support U_11_	None	51	− 0.0704	− 0.0389	− 0.0499
	shotcrete	52	− 0.0704	− 0.0389	− 0.0499
	Anchor	53	− 0.0936	− 0.0421	− 0.0695
	Steel arch	54	0.1576	− 0.1153	0.0600
	Loop excavation	55	− 0.0480	0.1180	0.0071

**Figure 4:**
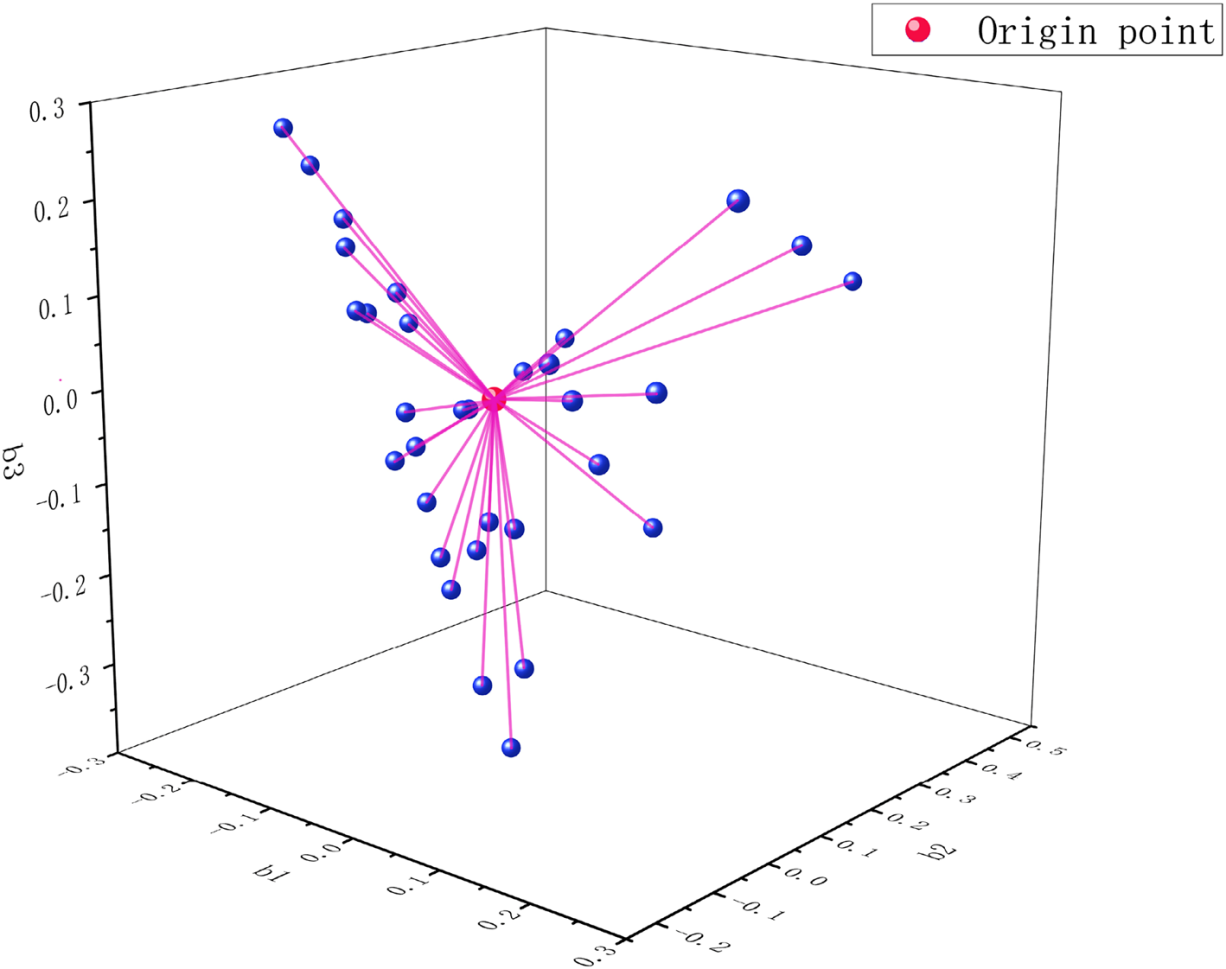
The distance of each category compared to the origin.

It can be seen from Table 3 that the correlation ratio of *λ*_1_ eigenvalue is 41%, and its trend change is the main macroscopic reflection of the influencing factors of tunnel collapse, and can be used as the main factor axis for screening sensitive factors. In order to more comprehensively reflect the information of the influencing factors, according to the previous restriction of *g*^*T*^*b* = *0*, the category scores *b*_1_, *b*_2_, *b*_3_ corresponding to *λ*_1_, *λ*_2_, *λ*_3_ are regarded as the spatial *X*, *Y*, and *Z* factor axes, and the zero point is establish as the origin of the spatial coordinate system, based on the spatial distance from the origin to perform category screening and classification (Fig. 4) (Table 4).

**Table 4:** Spatial distance statistics of influencing factor categories.

Category number	Spatial distance $\kappa$	Category number	Spatial distance $\kappa$	Category number	Spatial distance $\kappa$
1	0.0947	20	0.4959	39	0.2063
2	0.0875	21	0.0947	40	0.4959
3	0.2043	22	0.0922	41	0.1552
4	0.0947	23	0.3004	42	0.1497
5	0.0947	24	0.2652	43	0.0947
6	0.0747	25	0.3366	44	0.2119
7	0.0947	26	0.1634	45	0.3441
8	0.0947	27	0.1181	46	0.2128
9	0.2990	28	0.0947	47	0.1334
10	0.0947	29	0.3366	48	0.2096
11	0.1556	30	0.3717	49	0.4845
12	0.2096	31	0.0947	50	0.0947
13	0.4845	32	0.2447	51	0.0947
14	0.2916	33	0.0947	52	0.0947
15	0.0947	34	0.1412	53	0.1240
16	0.0947	35	0.4959	54	0.2043
17	0.2447	36	0.1612	55	0.1276
18	0.1975	37	0.1687		
19	0.2990	38	0.1486		

Since each item is a subset of each item, the final spatial distance of each item is obtained by the sum of the corresponding category distances (Table 5):

$$ \kappa_{ui} = \sum\limits_{{r_{1} = 1}}^{m} {\kappa_{{r_{i} }} } $$

**Table 5:** Spatial distance of each project.

Influencing factors	U_1_	U_2_	U_3_	U_4_	U_5_	U_6_	U_7_	U_8_	U_9_	U_10_	U_11_
Spatial distance $\kappa$	0.5758	0.6577	1.2360	1.2774	1.2759	1.0844	0.8523	1.1808	0.9556	1.1350	0.6451

Since the theory mainly considers the frequency of occurrence of each factor and the inherent meaning of each sample, the correlation between the categories of evaluation factors in practical engineering cannot be ignored^24–28^. Considering that the quantitative theory has more detailed classification scores for various categories, we use the Kendall algorithm to calculate the correlation between each factor. The core idea is to calculate the number of different pairs between two ordered sets.

Convert the original monitoring data of the tunnel in Table 1 into the spatial distance calculated in Sect. “Improvement steps”, as shown in Table 6 below.

**Table 6:** Spatial distance of influencing factors of each sample.

Serial number	U_1_	U_2_	U_3_	U_4_	U_5_	U_6_	U_7_	U_8_	U_9_	U_10_	U_11_
S1	0.2043	0.0747	0.1556	0.1975	0.0922	0.1634	0.1412	0.1687	0.1552	0.2128	0.0947
S2	0.2043	0.0947	0.4845	0.2990	0.3366	0.3366	0.1412	0.2063	0.2119	0.1334	0.0947
S3	0.2043	0.0947	0.2096	0.2990	0.2652	0.1181	0.1412	0.1486	0.1497	0.2096	0.0947
S4	0.0875	0.0747	0.1556	0.1975	0.0922	0.1634	0.1412	0.1687	0.2119	0.1334	0.1240
S5	0.0875	0.0747	0.1556	0.2447	0.3004	0.1634	0.2447	0.1486	0.3441	0.2128	0.1240
S6	0.0875	0.0747	0.2096	0.1975	0.2652	0.3717	0.1412	0.1687	0.1497	0.2096	0.1240
S7	0.0875	0.0747	0.1556	0.2447	0.3004	0.1634	0.2447	0.1486	0.1497	0.2128	0.1276
S8	0.0875	0.0747	0.2096	0.4959	0.3366	0.3366	0.4959	0.4959	0.1552	0.2096	0.1276
S9	0.0875	0.0747	0.1556	0.2447	0.0922	0.1181	0.2447	0.1612	0.1552	0.1334	0.1276
S10	0.0875	0.0747	0.2916	0.1975	0.2652	0.1181	0.1412	0.2063	0.1497	0.1334	0.1276

**Figure 5:**
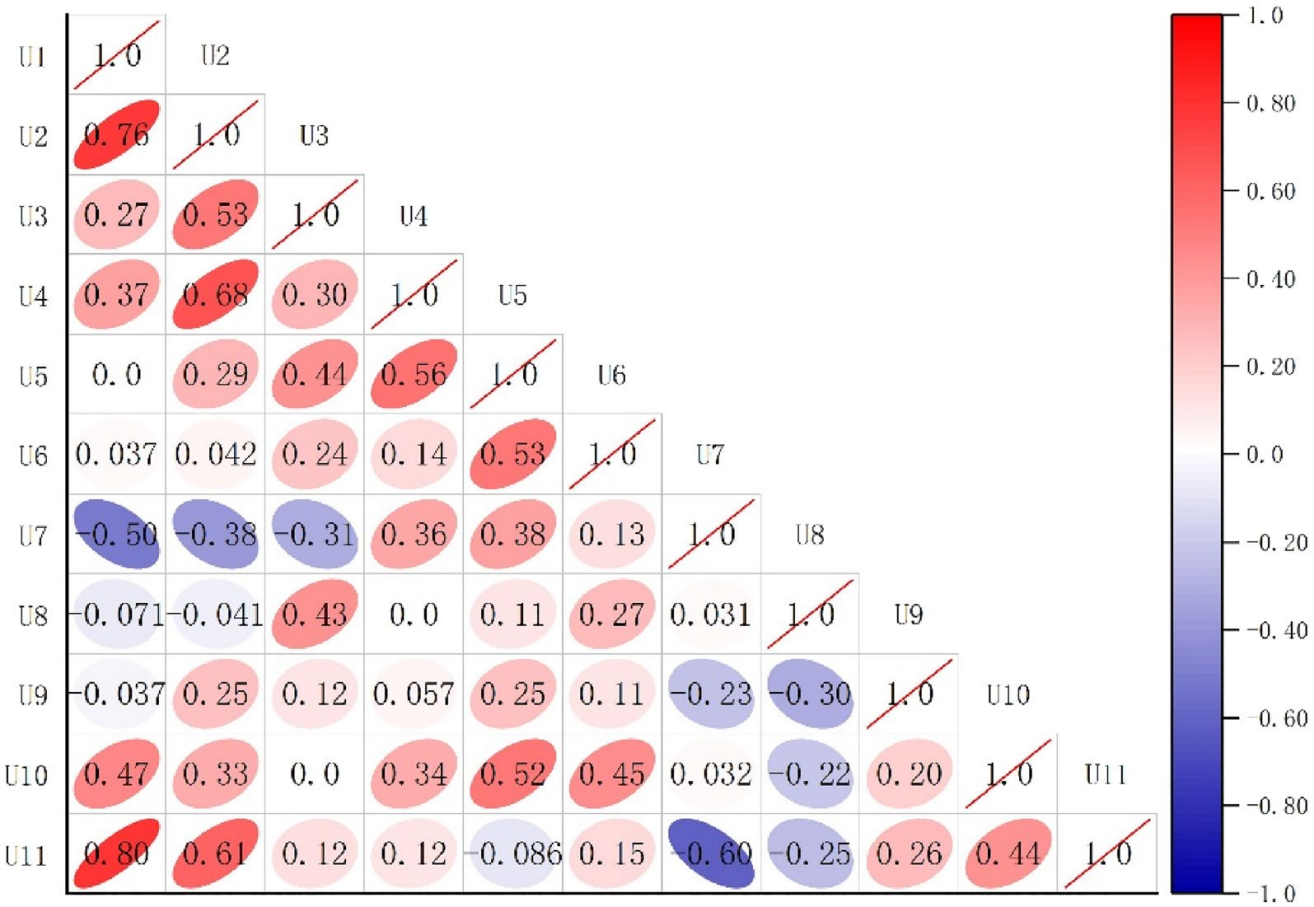
Correlation analysis between various influencing factors.

Equation (12) The Kendall algorithm is used to calculate the correlation between the evaluation factors as shown in the following Fig. 5:

The results are shown in Fig. 5: the correlation between the *U*_1_ (the area of equivalent cross-section) and the *U*_11_ (the method of support) is as high as 0.8, the correlation between the *U*_1_ (the area of equivalent cross-section) and the *U*_2_ (depth ratio) is also 0.76, and the correlation between *U*_2_ (depth ratio) and *U*_11_ (the method of support) also reached 0.61, and the comprehensive analysis was in line with the actual situation of tunnel construction.

**Figure 6:**
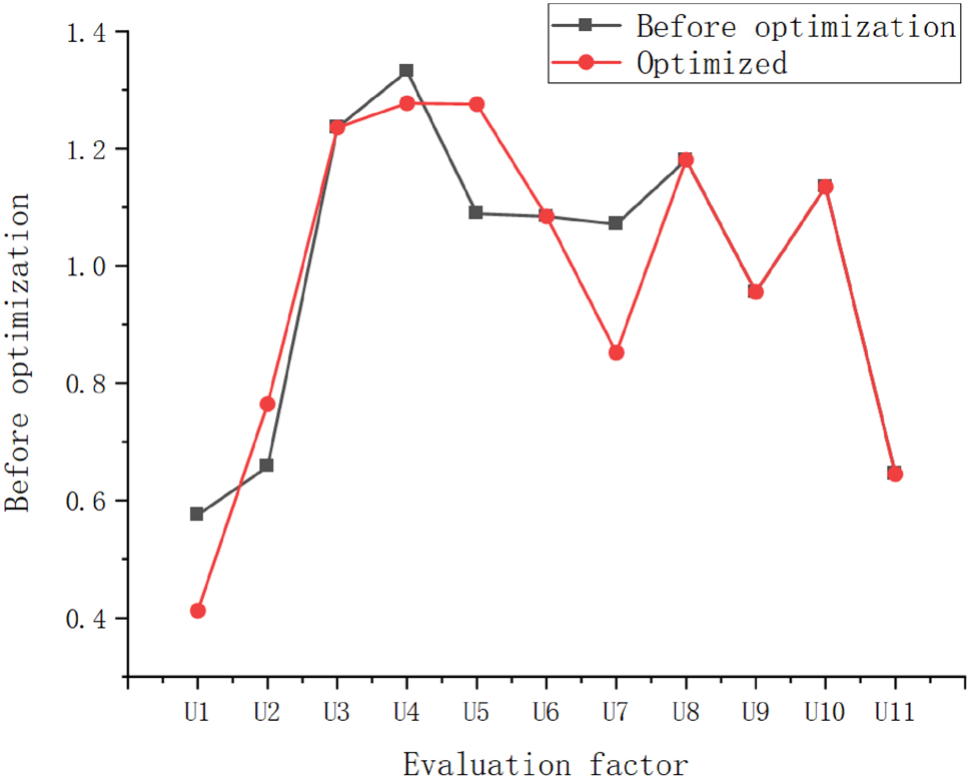
Comparison of spatial distance κ before and after optimization.

To this end, the correlation feedback is used to adjust the spatial distance (i.e. the weight), and according to the Rocchio Eq. (13), Fig. 6:

It can be seen that after optimization and adjustment, based on the characteristics of the case problem, several evaluation factors with strong correlation with other evaluation factors fluctuate greatly, indicating that reasonable weights should be allocated according to the strength of correlation within the evaluation factor group.

The spatial distance of each item is normalized to obtain the theory of quantification III weight of each evaluation factor (Table 7):

$$ \alpha_{j} = {{\kappa_{uj} } \mathord{\left/ {\vphantom {{\kappa_{uj} } {\sum\limits_{j = 1}^{11} {\kappa_{uj} } }}} \right. \kern-\nulldelimiterspace} {\sum\limits_{j = 1}^{11} {\kappa_{uj} } }} $$

**Table 7:** TQ-III weights.

Influencing factors	U_1_	U_2_	U_3_	U_4_	U_5_	U_6_	U_7_	U_8_	U_9_	U_10_	U_11_
TQ-III weights	0.0381	0.0707	0.1142	0.1181	0.1179	0.1002	0.0788	0.1091	0.0883	0.1049	0.0596

### Coupling weights

According to the calculation process given by the Entropy Weight method in Sect. “Coupling weights”, the original data is dimensionless by Eq. (14), as shown in Table 8:

**Table 8:** Dimensionless data.

Serial number	U_1_	U_2_	U_3_	U_4_	U_5_	U_6_	U_7_	U_8_	U_9_	U_10_	U_11_
S1	0.320	− 0.896	0.001	− 0.374	− 0.809	0.039	− 0.326	− 0.799	0.101	0.101	0.701
S2	0.320	− 0.249	0.418	− 0.074	− 0.219	0.386	− 0.254	− 0.299	0.701	0.701	0.701
S3	0.320	− 0.179	0.334	− 0.199	− 0.449	0.263	− 0.290	− 0.549	0.301	0.501	0.701
S4	0.258	− 2.839	0.001	− 0.274	− 0.879	0.116	− 0.308	− 0.799	0.701	0.301	0.501
S5	0.258	− 1.729	0.001	− 0.504	− 0.709	0.155	− 0.690	− 0.619	0.901	0.101	0.501
S6	0.258	− 2.719	0.251	− 0.274	− 0.449	0.424	− 0.435	− 0.799	0.301	0.501	0.501
S7	0.159	− 1.999	0.001	− 0.699	− 0.699	0.078	− 0.726	− 0.699	0.301	0.101	0.901
S8	0.159	− 2.239	0.251	− 0.019	− 0.139	0.347	− 0.217	− 0.199	0.101	0.501	0.901
S9	0.159	− 2.749	0.001	− 0.539	− 0.770	0.247	− 0.690	− 0.899	0.101	0.301	0.901
S10	0.159	− 2.759	0.668	− 0.274	− 0.409	0.270	− 0.435	− 0.399	0.301	0.301	0.901

The difference coefficient is calculated by Eqs. (15) and (16), and then brought into Eq. (17) to obtain its weight value under the Entropy Weight method, as shown in Table 9:

**Table 9:** EW weights.

Influencing factors	U_1_	U_2_	U_3_	U_4_	U_5_	U_6_	U_7_	U_8_	U_9_	U_10_	U_11_
EW weights	0.0867	0.0766	0.0763	0.0722	0.0874	0.0942	0.1256	0.0784	0.1142	0.0942	0.0942

According to the calculation process given by the Analytic Hierarchy Process Sect. “Coupling weights”, the judgment matrix M_1_, M_2_ is constructed for the two levels of geological condition and construction disturbance by Eq. (18):

$$ M_{1} = \left( {\begin{array}{*{20}c} 1 & 1 & 1 & {1/2} & {1/2} & {1/3} & {1/3} \\ 1 & 1 & 1 & 1 & 1 & {1/3} & {1/3} \\ 1 & 1 & 1 & {1/2} & {1/2} & {1/2} & {1/2} \\ 2 & 1 & 2 & 1 & 1 & 1 & 1 \\ 2 & 1 & 2 & 1 & 1 & 1 & 1 \\ 3 & 3 & 2 & 1 & 1 & 1 & 1 \\ 3 & 3 & 2 & 1 & 1 & 1 & 1 \\ \end{array} } \right) $$

$$ M_{2} = \left( {\begin{array}{*{20}c} 1 & 1 & {1/2} & {1/2} \\ 1 & 1 & 1 & {1/2} \\ 2 & 1 & 1 & 1 \\ 2 & 2 & 1 & 1 \\ \end{array} } \right) $$

According to the judgment matrix, the largest eigenvalue and the eigenvector of the matrix are calculated, and then bring them into the Eqs. (19) and (20) to pass the consistency test (CI =  < 0.1), so as to obtain the AHP weight value of each evaluation index. as shown in Table 10.

**Table 10:** AHP weights.

Analytic hierarchy process (AHP) results						
	Influencing factors	Feature vector	AHP weights per level	AHP weights	Largest characteristic root	CI value
Geological condition	U1	0.5993	0.0803	0.0511	7.1688	0.0281
	U2	0.7306	0.0979	0.0623		
	U3	0.673	0.0902	0.0574		
	U4	1.219	0.1633	0.1039		
	U5	1.219	0.1633	0.1039		
	U6	1.5112	0.2025	0.1289		
	U7	1.5112	0.2025	0.1289		
Construction disturbance	U1	0.7071	0.1703	0.0737	4.0606	0.0202
	U6	0.8409	0.2026	0.0619		
	U2	1.1892	0.2865	0.1042		
	U11	1.4142	0.3407	0.1239		

After calculating the weight value of each hierarchy, the AHP weights are obtained by coupling the two hierarchies in a ratio of 7/4.

**Figure 7:**
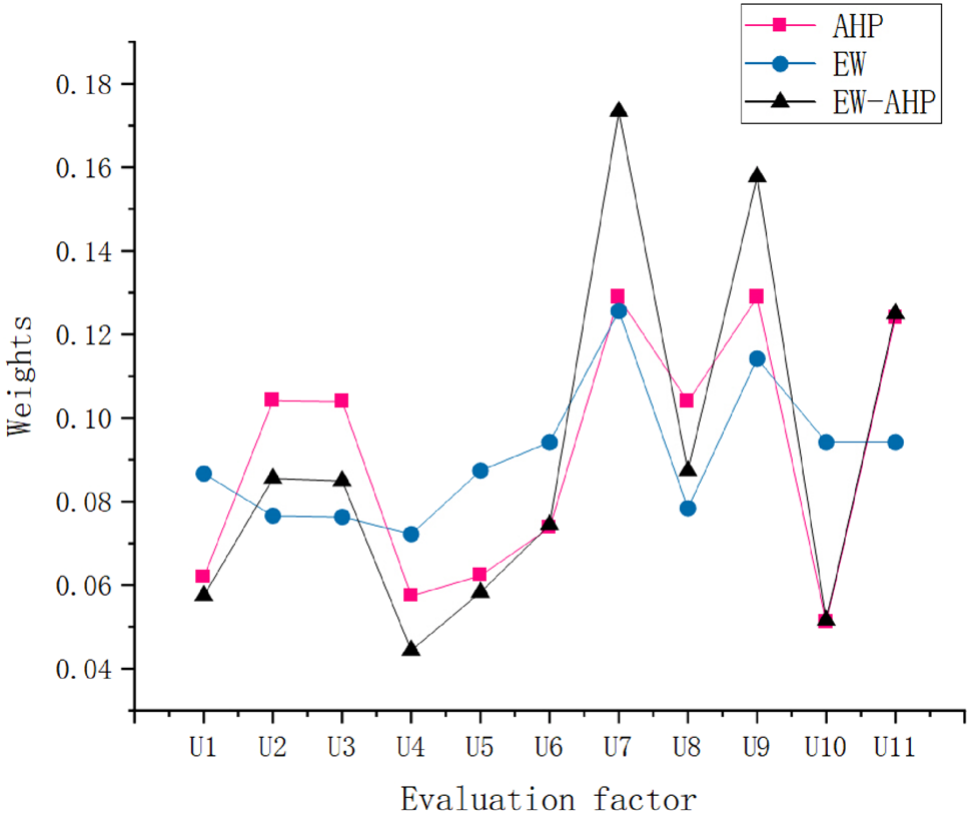
EW-AHP coupling weights.

After calculating the weight of the entropy weight method (EW) and the weight of the analytic hierarchy process (AHP), the EW-AHP coupling weight is obtained using the multiplicative synthesis normalization Eq. (21), which is used as the initial weight (as shown in Fig. 7).

**Figure 8:**
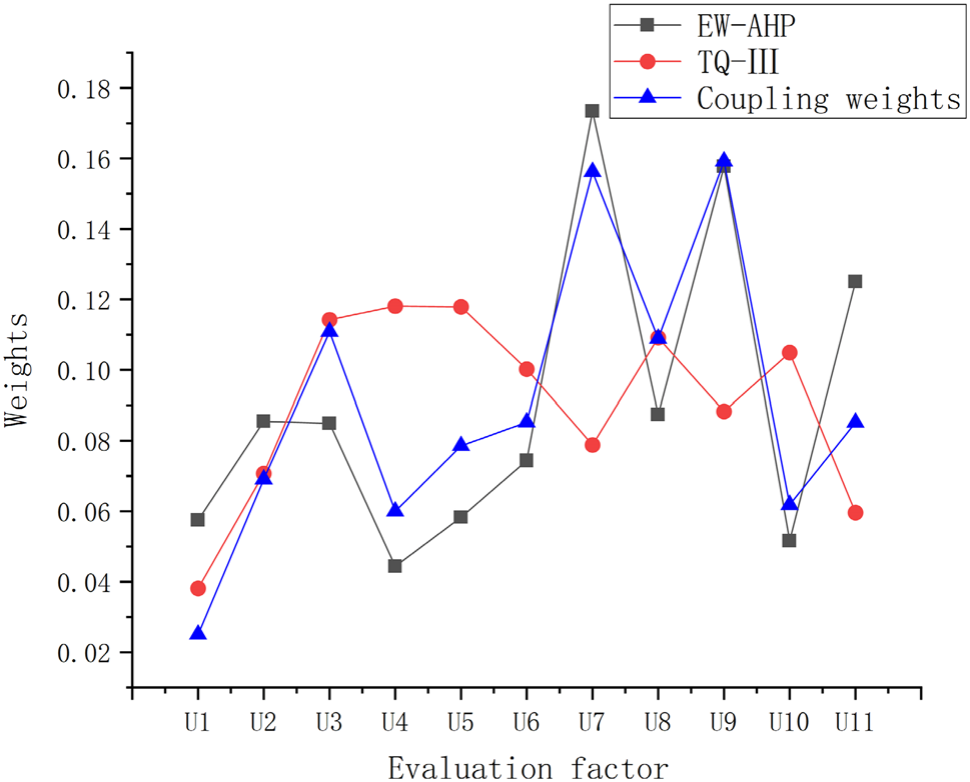
TQ-III and EW-AHP coupling weights.

Continue to use the multiplication synthesis normalization Eq. (21) to couple the EW-AHP initial weight and the TQ-III weight calculated earlier to obtain the final synthesis weight (as shown in Fig. 8).

### Fuzzy comprehensive evaluation

In this paper, referring to the method of Cao et al.^42^, a fuzzy comprehensive evaluation model of tunnel collapse risk is established, and it is applied to the assessment of the collapse risk of the Ka-shuang diversion tunnel. The 11 evaluation factors selected above are taken as the factor set in the fuzzy comprehensive evaluation, and recorded as *U* = {*U*_1_, *U*_2_, *U*_3_, *U*_4_, *U*_5_, *U*_6_, *U*_7_, *U*_8_, *U*_9_, *U*_10_, *U*_11_} (Table 1 in Sect. “Overview of research object’ sproject and geology”). And a comment set with 5 risk levels *V* = {*v*_1_, *v*_2_, *v*_3_, *v*_4_, *v*_5_} is formulated at the same time (Table 2 in Sect. “Overview of research object’ sproject and geology”) .

After determining the evaluation original data and the risk level limit of each factor, the position parameter *a*_*i*_ and the shape parameter *σ* of the normal distribution are calculated by Eqs. (27) and (28), as shown in Table 11:

**Table 11:** Normal distribution constant.

	Level I		Level II		Level III		Level IV		Level V	
	ai	σ	ai	σ	ai	σ	ai	σ	ai	σ
U_1_	10	12	32.5	15	57.5	15	85	30	135	18
U_2_	0.5	0.6	1.75	0.9	3.5	1.2	5.75	1.5	8.5	1.8
U_3_	0	0	10	12	25	6	40	12	60	12
U_4_	5	6	30	24	75	30	125	30	175	30
U_5_	0.125	0.15	0.375	0.15	0.625	0.15	0.825	0.15	0.95	0.06
U_6_	0.5	0.3	1.025	0.03	1.075	0.03	1.15	0.06	1.25	0.06
U_7_	0.75	0.9	2	0.6	3	0.6	4	0.6	5	0.6
U_8_	0.1	0.12	0.35	0.18	0.625	0.15	0.825	0.09	0.95	0.06
U_9_	0.1	0.12	0.3	0.12	0.5	0.12	0.7	0.12	0.9	0.12
U_10_	0.1	0.12	0.3	0.12	0.5	0.12	0.7	0.12	0.9	0.12
U_11_	0.1	0.12	0.3	0.12	0.5	0.12	0.7	0.12	0.9	0.12

Among them, since *U*_9_, *U*_10_, and *U*_11_ are qualitative variables, they are divided into equal proportions from 0 to 1 when calculating the membership degree.

After calculating the position parameter *a*_*i*_ and the shape parameter *σ*, the membership matrix B of each tunnel sample is calculated by Eqs. (22)–(26). Combined with the coupling weight W calculated in Sect. “Coupling weights”, the final risk rating of the 10 tunnel samples is calculated by Eq. (30).

Due to the large number of tunnel samples and the complicated calculation, this paper takes the tunnel sample *S1* as an example, and calculates the fuzzy judgment matrix *B*_1_ of *S1* by Eq. (22)–(26) as:

$$ B_{1} = \left( {\begin{array}{*{20}c} 0 & {0.35} & {0.66} & {0.21} & 0 \\ 1 & 0 & 0 & 0 & 0 \\ 1 & {0.3} & 0 & 0 & 0 \\ 0 & {0.06} & 1 & 0 & 0 \\ 0 & 1 & {0.22} & 0 & 0 \\ {0.5} & 0 & 0 & 0 & 0 \\ 0 & 0 & 0 & {0.89} & 1 \\ 0 & {0.93} & {0.73} & 0 & 0 \\ 1 & {0.21} & 0 & 0 & 0 \\ 1 & {0.21} & 0 & 0 & 0 \\ 0 & 0 & {0.06} & 1 & 0 \\ \end{array} } \right) $$

After calculating the membership matrix *B*_1_ of *S1*, combined with the coupling weight W obtained above, the fuzzy comprehensive vector $A_{1} = \left( {0.4436 \, 0.2719 \, 0.1783 \, 0.2295 \, 0.1562} \right)$ of *S1* is obtained by Eq. (30). After normalization, the membership degree $A_{1}^{\prime } = \left( {0.3467 \, 0.2125 \, 0.1394 \, 0.1793 \, 0.1221} \right)$ corresponding to each risk level is obtained.

According to the principle of maximum membership degree, the maximum membership degree in $A_{1}^{^{\prime}}$ is 0.3467, the corresponding risk level is level *I* (no risk), and the degree of membership in level *II* (slight risk) also reaches 0.2125, therefore, the possibility of S1 collapsing is very small. The remaining 9 tunnel samples were evaluated by the same method, and the final results are shown in Table 12.

**Table 12:** Comparison of evaluation results with field investigation.

Tunnel sample serial number	Fuzzy synthesis vector					Risk level (this method)	Risk level (Thesis^9^)	Site investigation results (whether a collapse occurred)
	Level I	Level II	Level III	Level IV	Level V			
S1	0.3467	0.2125	0.1394	0.1793	0.1221	I	I	×
S2	0.0000	0.0393	0.3143	0.4628	0.1835	IV	IV	√
S3	0.0086	0.2644	0.2938	0.3755	0.0577	IV	IV	√
S4	0.2105	0.2916	0.1627	0.2873	0.0480	II	I	×
S5	0.2452	0.2933	0.2838	0.0273	0.1505	II	I	×
S6	0.0722	0.3521	0.2797	0.2135	0.0825	II	III	×
S7	0.3286	0.4414	0.1474	0.0065	0.0760	II	I	×
S8	0.2276	0.1202	0.0664	0.1943	0.3915	V	V	√
S9	0.3675	0.4803	0.0710	0.0046	0.0767	II	I	×
S10	0.0773	0.2442	0.2366	0.3621	0.0797	IV	IV	√

It can be seen from Table 12 that:

1. There is one sample with the risk possibility of tunnel collapse level *I* (no risk), which is S1.The results field investigation shows that the surrounding rocks there are of high quality, with few fissures, complete and stable as a whole, and the walls of the caves are relatively smooth after excavation^9,41^.
2. There are four samples with the risk possibility of tunnel collapse level *II* (slight risk), namely S5, S6, S7 and S9. The results of field investigation show that the surrounding rock at S5 is of high quality grade, with few cracks, the whole is complete and stable, and the wall of the cave is relatively smooth after excavation; The surrounding rock at S6 has relatively good integrity and stability, and there is a slight local rockfall. The surrounding rock fractures at S7 and S9 are not well developed, and the overall stability and integrity are good^9,41^.
3. It is worth noting that *S4* is classified as level *IV* according to the maximum membership degree, but the membership degrees of level *II* (slight risk) and level *IV* (slight collapse) are very close and the overall trend is biased towards the low risk area. Therefore, the risk possibility of the S4 is considered to be level *III* (high risk) as a compromise. The actual investigation on the site shows that the surrounding rock fissures are not well developed, the overall stability and integrity are good, and local rock falls slightly too^9,41^.
4. There are three samples with the risk possibility of tunnel collapse level *IV* (slight collapse), which are S2, S3, and S10. The actual investigation on the site shows that the surrounding rock at S2 has poor integrity and stability, showing a block-cracked structure. The vault collapsed along the structural plane, and the blocks fell off seriously, but no large-scale collapse occurred. The surrounding rock fissures at S3 were strongly cut, the fissure plane is smooth, and the vault collapses seriously along the structural plane. The f 51 torsional fault is developed at the bottom of the arch at S10. The rock mass in the fault is mylonite and fault gouge, showing a fractured structure and serious rockfall^9,41^.
5. The tunnel collapse risk possibility level of *V* (severe collapse) is S8. The actual investigation on site shows: The surrounding rock of the tunnel vault at S8 slumped severely, and the length of the collapse with the development direction of the fissure was larger, and the scale of the collapse at the tuff with smaller thickness was larger, and the height reached 0.5 m^9,41^.

By analyzing the evaluation results of 10 sample tunnels, the results made by this method are in line with the actual situation of the project, and are basically consistent with the evaluation results made by other methods. The evaluation results not only answer the question of whether the tunnel collapses, but also describe the level of each risk possibility in detail.

## Discussion

Combined with project examples, from the comparison of evaluation results, we found that the results of this paper are slightly different from other literature evaluation results^9^ (using EW-AHP weights), and analyzed the reasons:

1. In this paper, the weights of *U*_3_ (the width of the fracture zone) and *U*_4_ (the strength of uniaxial compression) calculated by TQ-III are 0.1142 and 0.1181, respectively, which are greatly improved compared to EW-AHP weights of 0.0848 and 0.0527. Analysis of the reasons shows that there are 5 regional fault zones and 72 secondary faults with obvious structural traces on the surface of the project. The fracture zone is generally 10–30 m wide, and the overall fault zone has a high degree of development^41^. The calculation process in this paper is based on the actual geological structure characteristics of the tunnel. Affected by this, the corresponding increase in the weight is more suitable for the actual geological situation.
2. The TQ-III weights of *U*_1_ (the area of equivalent cross-section) and *U*_11_ (the method of support) are 0.0381 and 0.0596, respectively, which are significantly reduced compared to EW-AHP weights of 0.0631 and 0.1160. Analysis of the reasons shows that the project is a water diversion tunnel with a smaller cross-sectional area than traditional highway tunnels, and the disturbance to surrounding rock during excavation is correspondingly reduced. In addition, small-sized tunnels have lower requirements for support methods. Therefore, the importance of reducing *U*_1_ and *U*_11_ is in line with the actual tunnel construction status, reflecting the adaptability of this method.
3. The TQ-III weight of *U*_7_ (the grade of surrounding rock) is 0.0788, which is significantly lower than the EW-AHP weight of 0.1772. Affected by the correlation analysis, as shown in Fig. 4, the correlation between *U*_7_ and *U*_11_ is − 0.6, so there is a corresponding decreasing trend. And the overall stability of the rock mass in the cave is good, the uniaxial saturated compressive strength is 15 ~ 140 MPa, and the surrounding rock grades are mostly II and III^41^. Therefore, the less weight assigned to *U*_7_ is in line with the geological characteristics of the project area.
4. The weight of *U*_9_ (the groundwater condition) TQ-III is 0.0883, which is significantly lower than that of EW-AHP, which is 0.1709. Combined with the general situation of the project area, because the research object is located on the edge of the desert, the surface water is very poor, and the bedrock fissure water is the main type of groundwater, and the water volume is weak. The results of drilling water pumping test by Deng et al.^39^ showed that the surrounding rock of the tunnel belongs to the micro-level micro-permeable layer. Therefore, assigning a lower weight to *U*_9_ is in line with the actual geological conditions of the project area.

To sum up, the mathematical meaning of the weights calculated based on TQ-III is that less weights are assigned to evaluation factors that appear less frequently, and more weights are assigned to frequently appearing factors. In practical projects, some influencing factors may have high weights, but they appear less or not even in the project. Therefore, reducing their weights and assigning them to other influencing factors can improve the accuracy of the evaluation.

In view of the fact that the test data source of this case is a water diversion tunnel with a cross-section of 7.1 m, the application effect on other sizes or types of tunnels needs to be verified. Through the analysis of the selected 11 evaluation factors, the evaluation process considers U_1_ (the area of equivalent cross-section) and other 10 evaluation factors commonly used in various types of tunnels, so the theoretically analysis, this method is also applicable to other tunnels of different sizes and types. Therefore, in the following research, the author tries to continue to optimize the theory and expand the scope of application of the method.

## Conclusion

Considering the complexity and subjectivity of multiple decision-making problems in tunnel risk assessment, this paper proposes a tunnel collapse risk classification method based on the Improved Theory of Quantification III coupling weight and the Fuzzy Comprehensive Evaluation Method. The new method calculates the weights by uniformly converting the quantitative and qualitative data of tunnel monitoring into quantitative scores using the Improved Theory of Quantification III, and improves the accuracy by coupling commonly used subjective and objective weights. The membership degree of each evaluation factor and the tunnel risk level is calculated according to the normal distribution function, so as to comprehensively judge the collapse risk level of each tunnel sample. The new method has obtained the following conclusions in the application of the Ka-shuang diversion tunnel.

1. This paper uses the Theory of Quantification III to establish a reflection matrix for tunnel monitoring data, thereby converting qualitative data into quantitative data, avoiding the subjectivity of the assignment method. And after the improvement, the correlation between the evaluation factors is considered, and the accuracy of the evaluation results is improved.
2. The case vertification shows that the weights determined by this method are based on the actual project monitoring data, change accordingly with the structural characteristics of the project area, and have the characteristics of practicability and flexibility.
3. The reliability of the method is verified by comparing the evaluation results of 10 tunnel samples with the status quo of project area.

## Data Availability

All data generated or analyzed during this study are included within the article.

## References

[1] Wang X, Lai J, Qiu J, Xu W, Wang L, Luo Y (2020). Geohazards, reflection and challenges in mountain tunnel construction of China: A data collection from 2002 to 2018. Geomat. Nat. Haz. Risk.

[2] Marzouk M, Mohamed B (2019). Integrated agent-based simulation and multi-criteria decision making approach for buildings evacuation evaluation. Saf. Sci..

[3] Kim JH, Kim CY, Lee SS, Lee JH (2017). A study on influence factors for tunnel collapse risk analysis using Delphi method. J. Eng. Geol..

[4] Li SC, Wu J (2019). A multi-factor comprehensive risk assessment method of karst tunnels and its engineering application. Bull. Eng. Geol. Env..

[5] Ou GZ, Jiao YY, Zhang GH, Zou JP, Tan F, Zhang WS (2021). Collapse risk assessment of deep-buried tunnel during construction and its application. Tunn. Undergr. Space Technol..

[6] Chu H, Xu G, Yasufuku N, Yu Z, Liu P, Wang J (2017). Risk assessment of water inrush in karst tunnels based on two-class fuzzy comprehensive evaluation method. Arab. J. Geosci..

[7] Dai CQ, Zhao ZH (2015). Fuzzy comprehensive evaluation model for construction risk analysis in urban subway. Int. J. Model. Simul. Sci. Comput..

[8] Gao CL, Li SC, Wang J, Li LP, Lin P (2018). The risk assessment of tunnels based on grey correlation and entropy weight method. Geotech. Geol. Eng..

[9] Zhai, Q. Risk assessment of tunnel collapse by EW-AHP and unascertained measure theory, Lanzhou Jiaotong University, Lanzhou China. 10.13578/j.cnki.issn.1671-1556.2020.05.014 (2020).

[10] Mahdevari S, Shahriar K, Sharifzadeh M, Tannant DD (2017). Stability prediction of gate roadways in longwall mining using artificial neural networks. Neural Comput. Appl..

[11] He L, Tang T, Hu Q, Cai Q, Li Z, Tang S, Wang Y (2021). Integration of interpretive structural modeling with fuzzy Bayesian network for risk assessment of tunnel collapse. Math. Probl. Eng..

[12] Mahdevari S, Khodabakhshi MB (2021). A hybrid PSO-ANFIS model for predicting unstable zones in underground roadways. Tunn. Undergr. Space Technol..

[13] Zhou J, Zhu S, Qiu Y, Armaghani DJ, Zhou A, Yong W (2022). Predicting tunnel squeezing using support vector machine optimized by whale optimization algorithm. Acta Geotech..

[14] Wu Z, Zou S (2020). A static risk assessment model for underwater shield tunnel construction. Sādhanā.

[15] Lin CJ, Zhang M, Li LP, Zhou ZQ, Liu S, Liu C, Li T (2020). Risk assessment of tunnel construction based on improved cloud model. J. Perform. Constr. Facil..

[16] Wang X, Li S, Xu Z, Li X, Lin P, Lin C (2019). An interval risk assessment method and management of water inflow and inrush in course of karst tunnel excavation. Tunn. Undergr. Space Technol..

[17] Hong ES, Lee IM, Shin HS, Nam SW, Kong JS (2009). Quantitative risk evaluation based on event tree analysis technique: Application to the design of shield TBM. Tunn. Undergr. Space Technol..

[18] Vaurio JK (2010). Ideas and developments in importance measures and fault-tree techniques for reliability and risk analysis. Reliab. Eng. Syst. Saf..

[19] Zhang M (2022). Prediction of rockburst hazard based on particle swarm algorithm and neural network. Neural Comput. Appl..

[20] Li L, Zhang SX, Li SH, Qiang Y, Zheng Z, Zhao DS (2021). Debris flow risk assessment method based on combination weight of probability analysis. Adv. Civ. Eng..

[21] Li JX, Wang CM, Wang GC, Liu W (2010). Analysis of landslide influential factors and coupling intensity based on third theory of quantification. Chin. J. Rock Mech. Eng..

[22] Huang Z, Meng L, Huang X, Wang W (2016). Prediction of landslide volum based on quantitative theory and BP neural network. Bull. Soil Water Conserv..

[23] Zhang S, Sun J, Ma J (2016). A stady of rock mass landslide in red rock area of east sichuan province based on third theory of quantification. Bull. Soil Water Conserv..

[24] Palmes P, Pung HK, Gu T, Xue W, Chen S (2010). Object relevance weight pattern mining for activity recognition and segmentation. Pervasive Mob. Comput..

[25] Al-Mohamade A, Bchir O, Ben Ismail MM (2020). Multiple query content-based image retrieval using relevance feature weight learning. J. Imaging.

[26] Craswell, N., Robertson, S., Zaragoza, H., & Taylor, M. Relevance weighting for query independent evidence. In *Proceedings of the 28th Annual International ACM SIGIR Conference on Research and Development in Information Retrieval* 416–423. 10.1145/1076034.1076106 (2005).

[27] Yuan J, He L, Dragut EC, Meng W, Yu C (2017). Result merging for structured queries on the deep web with active relevance weight estimation. Inf. Syst..

[28] Dekant W, Bridges J, Scialli AR (2017). A quantitative weight of evidence assessment of confidence in modes-of-action and their human relevance. Regul. Toxicol. Pharmacol..

[29] Abdi, H. in The Kendall rank correlation coefficient. *Encyclopedia of Measurement and Statistics* 508–510 (Sage, Thousand Oaks, CA, 2007).

[30] Lamontagne, L., & Guyard, A. B. Learning case feature weights from relevance and ranking feedback. In *The Twenty-Seventh International Flairs Conference* (2014).

[31] Ma Y, Shi T, Zhang W, Hao Y, Huang J, Lin Y (2019). Comprehensive policy evaluation of NEV development in China, Japan, the United States, and Germany based on the AHP-EW model. J. Clean. Prod..

[32] Geng Z, Liu F, Shang D, Han Y, Shang Y, Chu C (2021). Early warning and control of food safety risk using an improved AHC-RBF neural network integrating AHP-EW. J. Food Eng..

[33] Liu H, Wang X, Tan G, He X (2020). System reliability evaluation of a bridge structure based on multivariate copulas and the AHP–EW method that considers multiple failure criteria. Appl. Sci..

[34] Zhao J, Ji G, Tian Y, Chen Y, Wang Z (2018). Environmental vulnerability assessment for mainland China based on entropy method. Ecol. Ind..

[35] Taherdoost, H. Decision making using the analytic hierarchy process (AHP); A step by step approach. *Int. J. Econ. Manag. Syst*. https://ssrn.com/abstract=3224206 (2017).

[36] Xiaotong W (2020). Risk assessment of long-span tunnel based on AHP and expert scoring method. Mod. Tunn. Technol..

[37] Li X (2017). Fuzzy Mathematical Methods and Applications.

[38] Zhang C, Wu S, Wu J (2019). Study on risk assessment model of collapse during construction of mountain tunnel and its application. J. Safe. Sci. Technol..

[39] Deng M, Zhou X, Cui D, Ma Y, Li W, Xu M (2016). Key technologies for deep super-long water diversion tunnels: A case study of Ka-Shuang tunnel. Tunn. Constr..

[40] Zuo Z, Zhang JR, Fu HL, Peng WX (2019). Collapse analysis of tunnel portal based on catastrophe theory. Elec. J. Geotech. Eng.

[41] Report of engineering geological investigation of Ka-Shuang Tunnel. *Xinjiang Survey and Design Institute of Water Resources and Hydropower & Urumqi: Xinjiang Survey and Design Institute of Water Resources and Hydropower* (2015) (**in Chinese**).

[42] Cao, W., Li, S. & Zhang Y. A classification method of surrounding rock mass quality based on tunnel milling excavation construction adaptability. *Chin. J. Rock Mech. Eng.*10.13722/j.cnki.jrme.2019.0040.

[43] Shin HS, Kwon YC, Jung YS, Bae GJ, Kim YG (2009). Methodology for quantitative hazard assessment for tunnel collapses based on case histories in Korea. Int. J. Rock Mech. Min. Sci..

[44] JTG 3370.1-2018 Specification for Design of Highway Tunnels, Section 1, Civil Engineering (China Communications Press Co., Ltd.) (**in Chinese**).

